# Latent Profile Analysis of Sleep Patterns in Children With Autism Spectrum Disorder

**DOI:** 10.1002/pdi3.70030

**Published:** 2025-12-17

**Authors:** Ke Wang, Qiuhong Wei, Ting Yang, Feiyong Jia, Yan Hao, Jinchen Li, Jie Chen, Tingyu Li, Hongyu Chen, Ximing Xu

**Affiliations:** ^1^ Children Nutrition Research Center Children's Hospital of Chongqing Medical University National Clinical Research Center for Child Health and Disorders Ministry of Education Key Laboratory of Child Development and Disorders Chongqing Key Laboratory of Child Nuerodevelopment and Cognitive Disorders Chongqing China; ^2^ Big Data Center for Children's Medical Care Children's Hospital of Chongqing Medical University Chongqing China; ^3^ Department of Developmental and Behavioral Pediatrics The First Hospital of Jilin University Changchun China; ^4^ Department of Pediatrics Tongji Hospital Tongji Medical College Huazhong University of Science and Technology Wuhan China; ^5^ Bioinformatics Centre, National Clinical Research Centre for Geriatric Disorders, Department of Geriatrics Xiangya Hospital & The Key Laboratory of Pediatric Rare Diseases of the Ministry of Education School of Life Sciences Central South University Changsha China

## Abstract

Sleep disturbances significantly impact children with autism spectrum disorder (ASD), yet their heterogeneous manifestations remain poorly understood. This multicenter prospective cohort study employed latent profile analysis to identify distinct sleep phenotypes among 631 children with ASD (aged 3–6 years) and 768 typically developing (TD) controls across three Chinese cities representing Northern, Central, and Western regions. Analysis of Children's Sleep Habits Questionnaire data revealed three distinct sleep phenotypes based on optimal model fit determined by Bayesian information criterion. Compared to TD children who showed generally better sleep patterns with lower sleep onset delay and fewer disturbances overall, the ASD groups exhibited distinctive profiles: Cluster 1 (9.2%) exhibited severe disturbances across multiple domains (sleep anxiety, parasomnias, night wakings and sleep‐disordered breathing) and demonstrated the most severe autism symptoms; Cluster 2 (36.0%) presented a mixed profile with comparable bedtime resistance, sleep duration, and daytime sleepiness to TD children but elevated sleep‐disordered breathing; and Cluster 3 (54.8%) showed reduced sleep‐disordered breathing but elevated night waking and bedtime resistance. One‐year follow‐up data indicated that Cluster 3, characterized by mild sleep‐disordered breathing, showed significant improvements in core symptoms particularly in social cognition, communication, and motivation domains, whereas Clusters 1 and 2 demonstrated modest changes. These findings suggest that early identification of sleep phenotypes may predict treatment response and inform personalized intervention strategies. Our results underscore the importance of incorporating comprehensive sleep assessment and management into ASD care protocols.

## Graphical abstract

Children with autism spectrum disorder (ASD) exhibited three distinct sleep phenotypes. Cluster 3, characterized by mild sleep‐disordered breathing, showed significant improvements in core symptoms, whereas Clusters 1 and 2 demonstrated modest changes. Early identification of sleep phenotypes may predict treatment response and inform personalized intervention.

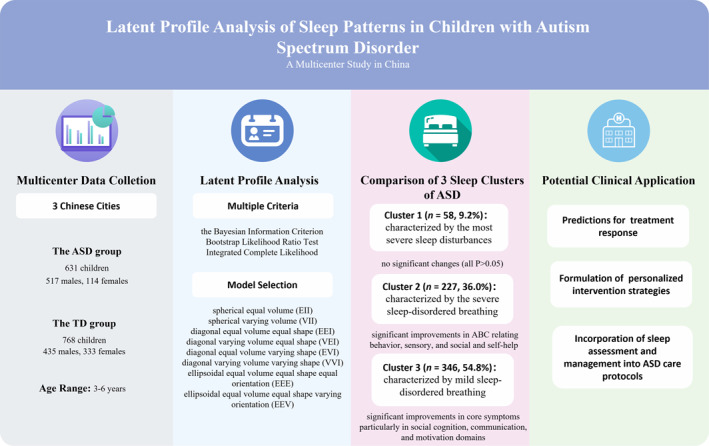

## 1 Introduction

Autism spectrum disorder (ASD) is a complex neurodevelopmental condition characterized by impairments in social communication and restricted repetitive behaviors [1]. Beyond its core symptoms, children with ASD often present with multiple comorbidities that not only impact their quality of life but may also interfere with treatment efficacy and increase disease burden [2].

Among these comorbidities, sleep disturbances represent one of the most prevalent symptoms in children with ASD. Research indicates that sleep problems affect 50%–80% of children with ASD [3, 4]. These sleep disturbances manifest across multiple dimensions, including difficulties with sleep onset, frequent night wakings, reduced sleep duration, sleep anxiety, and parasomnias [5, 6]. Adequate and quality sleep plays a crucial role in neurodevelopment, cognitive maturation, and emotional regulation during childhood [7]. Persistent sleep disruptions can impact brain structure and function, leading to dysfunction of neurotransmitter systems, metabolism, hormonal balance, and inflammatory processes, potentially contributing to the pathophysiology of neurodevelopment disorders [8].

Studies have revealed complex bidirectional interactions between sleep disturbances and core symptoms of ASD [9, 10, 11]. Sleep disorders are associated with social communication deficits, repetitive behaviors, and daytime functioning difficulties [12, 13]. Conversely, ASD symptoms (such as sensory hypersensitivity) demonstrate correlations with sleep problems [14, 15]. This reciprocal relationship suggests that sleep disturbances may not merely represent a comorbid condition but could be an integral component of ASD pathophysiology. Therefore, developing a deeper understanding of sleep patterns in children with ASD and their relationship with the core symptoms is crucial for improving patient outcomes.

However, sleep problems in children with ASD demonstrate significant heterogeneity [16]. Clinical observations reveal that different patients may exhibit varying types and severities of sleep disturbance, potentially reflecting distinct underlying neurobiological mechanisms [17]. Recent research approaches using single indicators or simple group comparisons have struggled to fully capture this heterogeneity and its clinical significance [18]. Furthermore, there is a relative paucity of longitudinal studies examining the long‐term impact of sleep problems on ASD outcomes, limiting our understanding of the value of sleep interventions in ASD treatment.

Latent profile analysis (LPA), a data‐driven statistical approach, offers a perspective for studying complex phenotypes by identifying subgroups with similar characteristics based on multiple continuous observational indicators [19, 20]. LPA's capacity to process multiple dimensional indicators simultaneously makes it particularly suitable for capturing the heterogeneous characteristics of complex disorders. This method has been applied in psychiatric research, demonstrating its utility in delineating cognitive subtypes in schizophrenia [21], characterizing symptom patterns in attention‐deficit/hyperactivity disorder [22], and identifying distinct clinical phenotypes in depressive disorders [23]. Given the multidimensional and heterogeneous nature of sleep problems in ASD, LPA provides an innovative approach for understanding this complex clinical issue.

In this study, we aimed to conduct a multicenter prospective cohort study to (1) identify distinct sleep phenotypes in children with ASD using LPA; (2) investigate the relationship between these sleep phenotypes and core symptoms of autism; and (3) examine the relationship between various sleep subtypes and ASD outcomes over a 1‐year follow‐up period.

## 2 Materials and Methods

### 2.1 Study Participants

This multicenter prospective cohort study was conducted from September 2019 to December 2023 across three Chinese cities selected to represent different geographical regions: Chongqing (Western region), Jilin (North region), and Wuhan (Central region). Children with ASD were recruited from developmental‐behavioral pediatric outpatient departments and specialized learning center, while typically developing (TD) children were recruited from local kindergartens and routine health check‐ups at pediatric clinics. The inclusion criteria for the ASD group consisted of age between 3 and 6 years, ASD diagnosis confirmed by experienced developmental‐behavioral pediatricians using DSM‐5 (Diagnostic and Statistical Manual of Mental Disorders, Fifth Edition) criteria, and primary caregiver's informed consent. For the TD group, inclusion criteria encompassed age between 3 and 6 years, absence of any neurodevelopmental disorders, no family history of ASD among first‐ and second‐degree relatives, and primary caregiver's informed consent. Both groups excluded children with severe physical illness, history of genetic or neurological conditions with known etiology or brain trauma affecting neurodevelopment, and incomplete questionnaire responses. This study was approved by the Medical Ethics Committee of Children's Hospital of Chongqing Medical University (Ethics approval number: 121‐1/2018) and registered with the Chinese Clinical Trial Registry (ChiCTR2000031194).

### 2.2 Clinical Assessment

Demographic information was collected via questionnaires, including gender, date of birth, medical history, and family history. Sleep patterns were assessed using the Children's Sleep Habits Questionnaire (CSHQ) [24], a caregiver‐reported instrument consisted of eight subscales evaluating the following sleep domains: bedtime resistance, sleep‐disordered breathing, daytime sleepiness, sleep anxiety, sleep duration, sleep onset delay, night wakings, and parasomnias. Each item was scored on a three‐point scale (1–3: rarely, sometimes, and usually), with higher scores indicating more severe sleep problems.

Core ASD symptoms were evaluated using three instruments. The Childhood Autism Rating Scale (CARS) [25], a 15‐item scale assessing autism severity, was scored 1–4 per item (total range: 15–60). The Social Responsiveness Scale (SRS) [26] is a 65‐item assessment tool that evaluates various aspects of social behavior across five domains: social awareness, social cognition, social communication, social motivation, and autistic mannerisms. Each item is rated on a 4‐point scale ranging from 0 (not true) to 3 (almost always true), yielding a total score range of 0–195, with higher scores indicating greater social impairment. The Autism Behavior Checklist (ABC) [27] consists of 57 items across five domains: (1) sensory behavior (responses to tactile, visual, auditory, and olfactory stimuli), (2) relating behavior (eye contact, social responsiveness, and ability to form relationships), (3) body and object use (stereotypical movements, unusual body postures, and atypical object manipulation), (4) language skills (receptive and expressive communication), and (5) social and self‐help skills (adaptive functioning in daily living activities). Each item has two options: “Yes” or “No”. Choosing “No” yields 0 points, while choosing “Yes” awards a fixed corresponding score (a predetermined value from 1 to 4 points, not a Likert scale rating). The total scores are ranging from 0 to 158. Developmental assessment utilized the Gesell Developmental Scale [28] and the Revised Children's Neuropsychological and Behavior Scale (CNBS‐R2016), yielding developmental quotients (DQs) across gross motor, fine motor, adaptive, language, and personal–social domains [29].

Primary caregivers completed the CSHQ for all participants. Children in the ASD group underwent additional assessments (CARS, ABC, SRS, and developmental evaluation) conducted by trained developmental‐behavioral pediatricians or psychological evaluators. For the longitudinal component of this study, only the Chongqing cohort participated in the follow‐up assessment after 1 year of behavioral intervention, with all baseline evaluations repeated at the follow‐up endpoint. The guardian chooses a qualified special education institution as the intervention site, and the institution constructs an individualized rehabilitation intervention system based on the clinical phenotypic characteristics of the child with ASD and the results of the standardized assessment.

### 2.3 Latent Profile Analysis

The study employed latent profile analysis to classify sleep characteristics among children with ASD [30]. The analysis was conducted using the mclust package in R version 4.4.2, modeling scores across the eight CSHQ sleep subscales. Model selection involved comparing different numbers of classes and covariance structures (spherical equal volume [EII], spherical varying volume [VII], diagonal equal volume equal shape [EEI], diagonal varying volume equal shape [VEI], diagonal equal volume varying shape [EVI], diagonal varying volume varying shape [VVI], ellipsoidal equal volume equal shape equal orientation [EEE], and ellipsoidal equal shape, equal orientation, varying volume [EEV]). The optimal classification was determined through multiple criteria: the Bayesian information criterion (BIC), with lower values indicating better model fit [31]; bootstrap likelihood ratio test (LRT), with 999 resampling iterations using the mclustBootstrapLRT function to assess statistical differences between models with adjacent class numbers; and integrated complete likelihood (ICL) to evaluate classification clarity. Subjects were assigned to their respective classes based on posterior probabilities derived from the optimal model selected through these comprehensive criteria.

### 2.4 Statistical Analysis

Statistical analyses were performed using R software (version 4.4.2). Continuous variables were presented as mean ± standard deviation or median (interquartile range) depending on their distribution characteristics, whereas categorical variables were expressed as frequencies (percentages). Comparisons between ASD and TD groups for CSHQ scores employed independent samples *t*‐tests or Mann–Whitney U tests for continuous variables and chi‐squared tests for categorical variables. CSHQ, CARS, ABC, and SRS scores across different sleep subtypes were compared using one‐way analysis of variance (ANOVA) or Kruskal–Wallis tests, with post hoc pairwise comparisons utilizing Bonferroni correction. Longitudinal data analysis employed paired *t*‐tests or Wilcoxon signed‐rank tests to compare baseline and follow‐up scores. All statistical tests were two‐sided, with statistical significance set at *p* < 0.05.

The study employed latent profile analysis to classify sleep characteristics among children with ASD [30]. The analysis was conducted using the mclust package in R version 4.4.2, modeling scores across the eight CSHQ sleep subscales. Model selection involved comparing different numbers of classes and covariance structures (EII, VII, EEI, VEI, EVI, VVI, EEE, and EEV). The optimal classification was determined through multiple criteria: BIC, with higher values indicating better model fit [31]; bootstrap LRT, with 999 resampling iterations using the mclustBootstrapLRT function to assess statistical differences between models with adjacent class numbers; and ICL to evaluate classification clarity. Subjects were assigned to their respective classes based on posterior probabilities derived from the optimal model selected through these comprehensive criteria.

## 3 Results

### 3.1 Study Population

**FIGURE 1:**
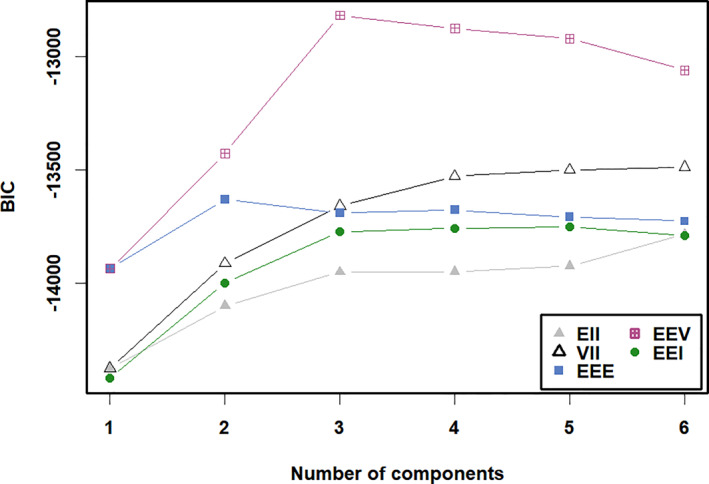
Bayesian information criterion (BIC) values for latent profile analysis models with different numbers of classes.

Between September 2019 and December 2023, we recruited 2063 children for this multicenter study. After excluding cases with missing regional information (*n* = 13), incomplete questionnaires (*n* = 101), and those not meeting age criteria of 3–6 years (*n* = 550), the final analysis included 1399 children. The ASD group comprised 631 children (mean age of 4.32 years; 517 males and 114 females), whereas the TD group included 768 children (mean age of 4.71 years; 435 males and 333 females). The geographical distribution encompassed Chongqing (*n* = 1013), Jilin (*n* = 307), and Wuhan (*n* = 79). Table 1 presents demographic characteristics and CSHQ subscale scores across eight sleep domains: bedtime resistance, sleep‐disordered breathing, daytime sleepiness, sleep anxiety, sleep duration, sleep onset delay, night wakings, and parasomnias. Additionally, preliminary analysis of the eight CSHQ subscales demonstrated homogeneous sleep pattern distributions across geographical locations (Supporting Information S1: Figure 1); given this consistency between the regions, data from all three sites were integrated into a unified dataset for subsequent statistical analyses.

**TABLE 1:** Demographic and sleep characteristics of ASD and TD groups.

Characteristics	ASD	TD
*N*	631	768
Age, mean (SD)	4.32 (0.94)	4.71 (1.01)
Gender, *n*		
Male	517	435
Female	114	333
CSHQ subscales, mean (SD)		
Bedtime resistance	12.14 (2.30)	11.36 (2.49)
Sleep‐disordered breathing	3.34 (0.70)	3.29 (0.65)
Daytime sleepiness	11.49 (2.71)	11.71 (2.83)
Night wakings	3.49 (0.89)	3.42 (0.90)
Parasomnias	8.56 (1.55)	8.48 (1.63)
Sleep anxiety	6.98 (1.72)	7.15 (1.81)
Sleep duration	4.41 (1.56)	4.44 (1.53)
Sleep onset delay	1.84 (0.73)	1.70 (0.73)

Abbreviations: ASD, autism spectrum disorder; CSHQ, Children's Sleep Habits Questionnaire; *n*, number; SD, standard deviation; TD, typically developing.

### 3.2 Identification of Latent Sleep Profiles

**FIGURE 2:**
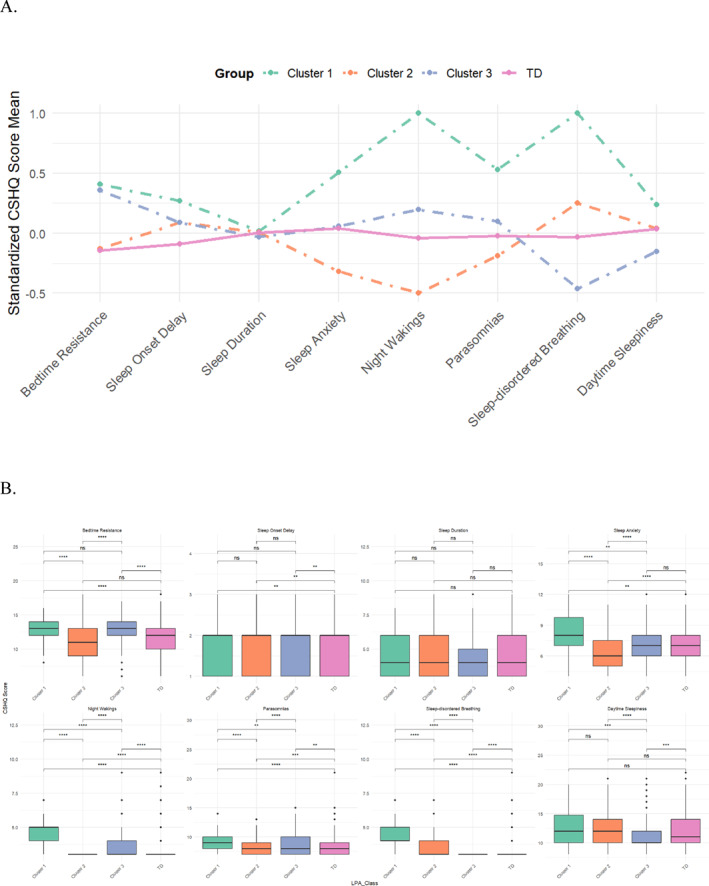
Children's Sleep Habits Questionnaire (CSHQ) scores across eight sleep domains for different latent profile analysis (LPA) of subgroups and typically developing (TD) children: (A) Comparison of standardized CSHQ mean scores; (B) comparison of raw CSHQ scores; *p < 0.05; **p < 0.01; ***p < 0.001; ****p < 0.00001; and ns, not significant.

Latent profile analysis of the eight CSHQ subscales revealed distinct sleep patterns among children with ASD. The EEV model (equal shape, equal orientation, and varying volume) yielded optimal fit with a three‐class solution based on BIC criteria (Figure 1) and ICL criteria (Supporting Information S1: Figure 2). Bootstrap LRTs (999 replications) demonstrated significant differentiation between class solutions: one versus two classes (LRTs = 746.7 and *p* < 0.001) and two versus three classes (LRTs = 848.9 and *p* < 0.001).

### 3.3 Sleep Phenotypes

LPA classification of the ASD group (*n* = 631) yielded three distinct clusters: Cluster 1 (*n* = 58, 9.2%), Cluster 2 (*n* = 227, 36.0%), and Cluster 3 (*n* = 346, 54.8%). Intercluster comparisons revealed distinctive sleep patterns (Figure 2). All ASD subgroups demonstrated significantly elevated sleep onset delay scores compared to TD controls (all *p* < 0.05), whereas sleep duration remained comparable.

Cluster 1 exhibited the most severe sleep disturbances, with significantly elevated scores in sleep anxiety, parasomnias, night wakings, and sleep‐disordered breathing compared to other ASD clusters and TD children (all *p* < 0.05). The most pronounced differences were observed in night wakings and sleep‐disordered breathing. Cluster 2 demonstrated a mixed profile relative to TD children: comparable scores in bedtime resistance, sleep duration, and daytime sleepiness (all *p* > 0.05); marginally lower scores in night wakings, parasomnias, and sleep anxiety (*p* < 0.05); and elevated scores in sleep‐disordered breathing (*p* < 0.05). Cluster 3, when compared to TD children, exhibited comparable scores in sleep duration and sleep anxiety (all *p* > 0.05), reduced sleep‐disordered breathing scores (*p* < 0.05), yet significantly higher scores in night wakings and bedtime resistance (all *p* < 0.05).

### 3.4 Neurodevelopmental Characteristics Across Sleep Patterns

**FIGURE 3:**
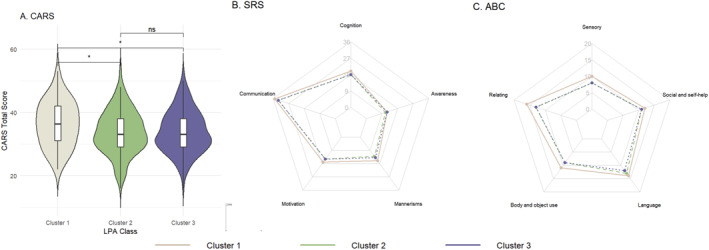
Comparison of autism spectrum disorder symptom severity across different sleep clusters: (A) Childhood Autism Rating Scale (CARS); (B) Social Responsiveness Scale (SRS); (C) Autism Behavior Checklist (ABC); *p < 0.05; and ns, not significant.

We compared the developmental behavioral characteristics of children with ASD across different sleep clusters, including ASD symptom severity and developmental levels. Regarding core autism symptoms (Figure 3), Cluster 1 demonstrated more severe symptoms overall, with a median CARS total score of 36.25, significantly higher than the median of 33 observed in both other clusters (both *p* < 0.05). Similarly, Cluster 1 showed elevated scores on the ABC scale with a median total score of 60.5, significantly higher than Cluster 2 (median = 52 and *p* < 0.05) and Cluster 3 (median = 49 and *p* < 0.05). The SRS total scores followed a similar pattern, with Cluster 1 showing a median score of 96, significantly higher than both Cluster 2 (median = 91 and *p* < 0.05) and Cluster 3 (median = 90 and *p* < 0.05).

In terms of specific subscale performance, the ABC scale revealed that Cluster 1 exhibited significantly higher scores in domains, such as body and object use (median = 9) and sensory processing (median = 10), compared to the other two clusters (all *p* < 0.05). On the SRS scale, Cluster 1 demonstrated relatively higher scores in social motivation (median = 17), cognition (median = 20), and mannerism (median = 16) compared to the other two clusters (all *p* < 0.05).

Regarding developmental levels, assessment using the children's developmental scale showed comparable developmental quotients across all three clusters. Language development quotients were consistently low across groups (medians of 46, 45, and 46, respectively), whereas gross motor development quotients were relatively higher (median range 68–73). Detailed results of developmental levels and autism symptoms are presented in Supporting Information S1: Figure 3.

### 3.5 Prognosis of Children With ASD Across Different Sleep Subgroups

We conducted a one‐year follow‐up of children with ASD from the Chongqing cohort only, with completion rates of 38 participants in Cluster 1, 141 in Cluster 2, and 196 in Cluster 3. Baseline comparison between children who completed the follow‐up and those who did not revealed no significant differences in demographic characteristics, autism symptom severity, or sleep patterns. Based on CSHQ tracking results, no significant differences were observed in eight domain scores between baseline and follow‐up across all three sleep subtypes (all *p* > 0.05, Supporting Information S1: Table 1). The longitudinal data indicated consistency in sleep pattern characteristics across all sleep subgroups of ASD throughout the follow‐up period.

**FIGURE 4:**
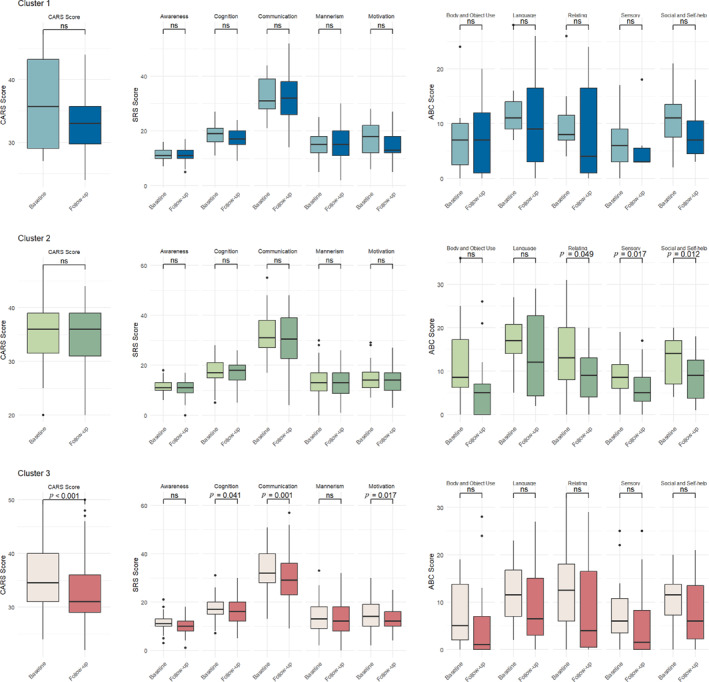
One‐year follow‐up outcomes across three sleep phenotypes in children with ASD. ABC, Autism Behavior Checklist; CARS, Childhood Autism Rating Scale; ns, not significant; SRS, Social Responsiveness Scale.

Comparisons of symptom improvement between baseline and follow‐up across the three subtypes are presented in Figure 4. Cluster 1 showed no significant changes in autism symptom severity as measured by CARS, ABC, and SRS (all *p* > 0.05). Cluster 2 demonstrated no significant changes in CARS and SRS scores but showed significant improvements in ABC relating behavior, sensory, and social and self‐help (all *p* < 0.05). Cluster 3 exhibited the significant reductions in CARS total scores (*p* < 0.001), SRS cognition, communication, and motivation (all *p* < 0.05).

## 4 Discussion

This large‐scale multicenter prospective cohort study provides novel insights into the heterogeneity of sleep disturbances in children with ASD through the application of latent profile analysis. Our findings reveal three distinct sleep phenotypes with differential associations to core autism symptoms and prognostic outcomes, suggesting the importance of sleep patterns in understanding ASD heterogeneity and potentially tailoring interventions.

This study identified three distinct sleep phenotypes highlighting the complex nature of sleep disturbances in ASD. Cluster 1 (9.2%) exhibited the most severe sleep problems across multiple dimensions, including sleep anxiety, parasomnias, night wakings, and sleep‐disordered breathing. This cluster also demonstrated the most severe autism symptoms across all assessment scales (CARS, ABC, and SRS), supporting the relationship between sleep disturbances and ASD core symptoms previously reported in the literature [32]. Clusters 2 (36.0%) and 3 (54.8%) presented with milder sleep problems but showed distinct patterns. This pattern differs from previous studies, such as Cohen et al.'s two‐cluster solution [33], potentially due to differences in population characteristics, assessment tools, and analytical methods. However, these studies collectively demonstrate the significant heterogeneity in sleep disturbances among children with ASD.

Our 1‐year follow‐up data revealed that Cluster 3 demonstrated significant improvements in core symptoms as measured by CARS scores, whereas Groups 1 and 2 showed relatively modest improvements. Further analysis indicated that Cluster 3's most distinctive characteristic was mild sleep‐disordered breathing. This finding aligns with previous research, demonstrating that sleep‐disordered breathing significantly correlates with cognitive function and behavioral problems in children with ASD [34, 35]. Our longitudinal follow‐up study provides additional and stronger evidence supporting this relationship. Moreover, a previous study confirmed that treatment of nocturnal breathing disorders can significantly enhance both sleep quality and daytime functioning in affected children [36]. These findings have several important clinical implications: (1) early identification of sleep phenotypes may help predict treatment response; (2) children with severe sleep disorders, particularly sleep‐disordered breathing, may require more aggressive comprehensive intervention strategies, such as early otolaryngology consultation and, when indicated, adenoidectomy; and (3) sleep management should be considered an integral component of early intervention programs for ASD.

Several limitations of this study warrant consideration. First, sleep assessment relied primarily on caregiver‐reported measures rather than objective sleep monitoring methods such as actigraphy or polysomnography. Future studies incorporating these objective measures could provide additional validation of the identified sleep phenotypes. Second, the relatively low follow‐up completion rate may affect the representativeness of our longitudinal analyses. Finally, our study did not systematically track specific interventions received by participants during the follow‐up period, limiting our ability to evaluate the impact of different treatment approaches on sleep patterns and core symptoms.

In conclusion, this study identified three distinct sleep phenotypes in children with ASD and demonstrated their differential associations with core symptoms and treatment outcomes over a 1‐year period. Our findings underscore the clinical utility of sleep phenotyping in ASD, suggesting that early identification of sleep patterns may inform prognosis and guide personalized intervention strategies. These results highlight the importance of incorporating comprehensive sleep assessment and management into standard ASD care protocols.

## Author Contributions

All authors contributed to the study conception and design. Data collection, and analysis were performed by Ke Wang. The first draft of the manuscript was written by Hongyu Chen, Qiuhong Wei, Ke Wang, and all authors commented the manuscript. All authors read and approved the final manuscript.

## Funding

The authors have nothing to report.

## Ethics Statement

This study was approved by the Medical Ethics Committee of Children's Hospital of Chongqing Medical University (Ethics approval number: 121‐1/2018) and registered with the Chinese Clinical Trial Registry (ChiCTR2000031194).

## Conflicts of Interest

The authors declare no conflicts of interest.

## Supporting information


Supporting Information S1

## Data Availability

Research data are not shared.
