# Supplementary material for: Latent Profile Analysis of Sleep Patterns in Children With Autism Spectrum Disorder
Source: Pediatr Discov. 2025 Dec 17;4(1):e70030. doi: 10.1002/pdi3.70030 (PMC13097561; doi:10.1002/pdi3.70030)
Supplement: Supplementary file 1 — Supporting Information S1 [file PDI3-4-e70030-s001.docx]

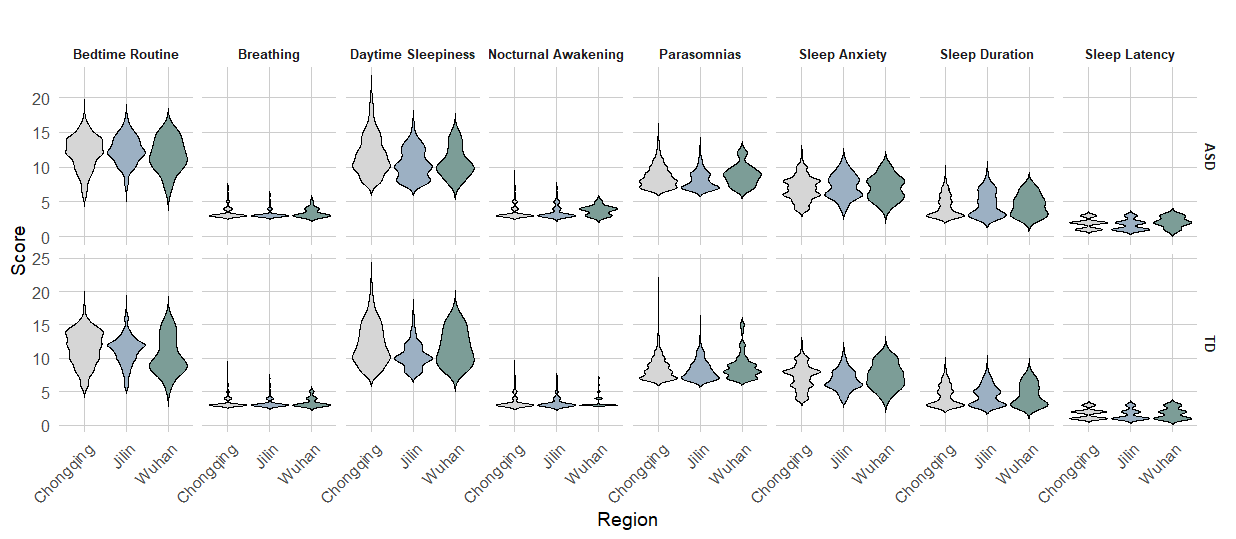
Supplementary Figure 1. Subscale Results of the Children's Sleep Habits Questionnaire (CSHQ) Across Three Regions


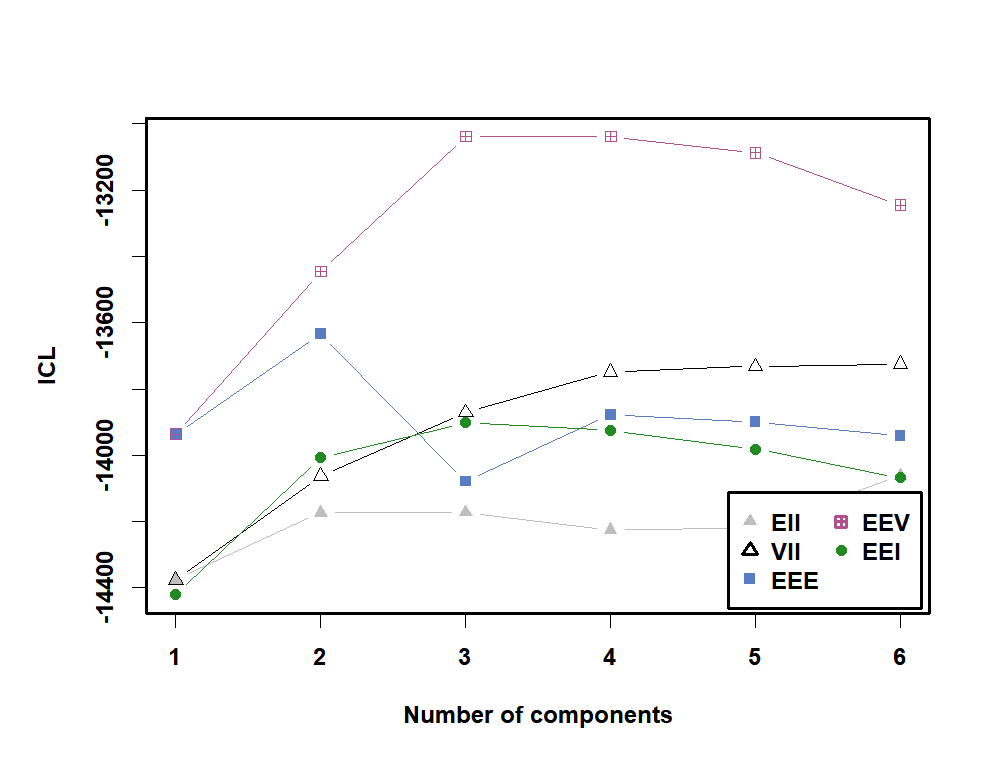


Supplementary Figure 2. Integrated Completed Likelihood (ICL) Values for Latent Profile Analysis Models with Different Numbers of Classes. The EVI model was not retained in the BIC comparison because it did not converge to a numerically stable solution for the given data, resulting in undefined BIC values.

A
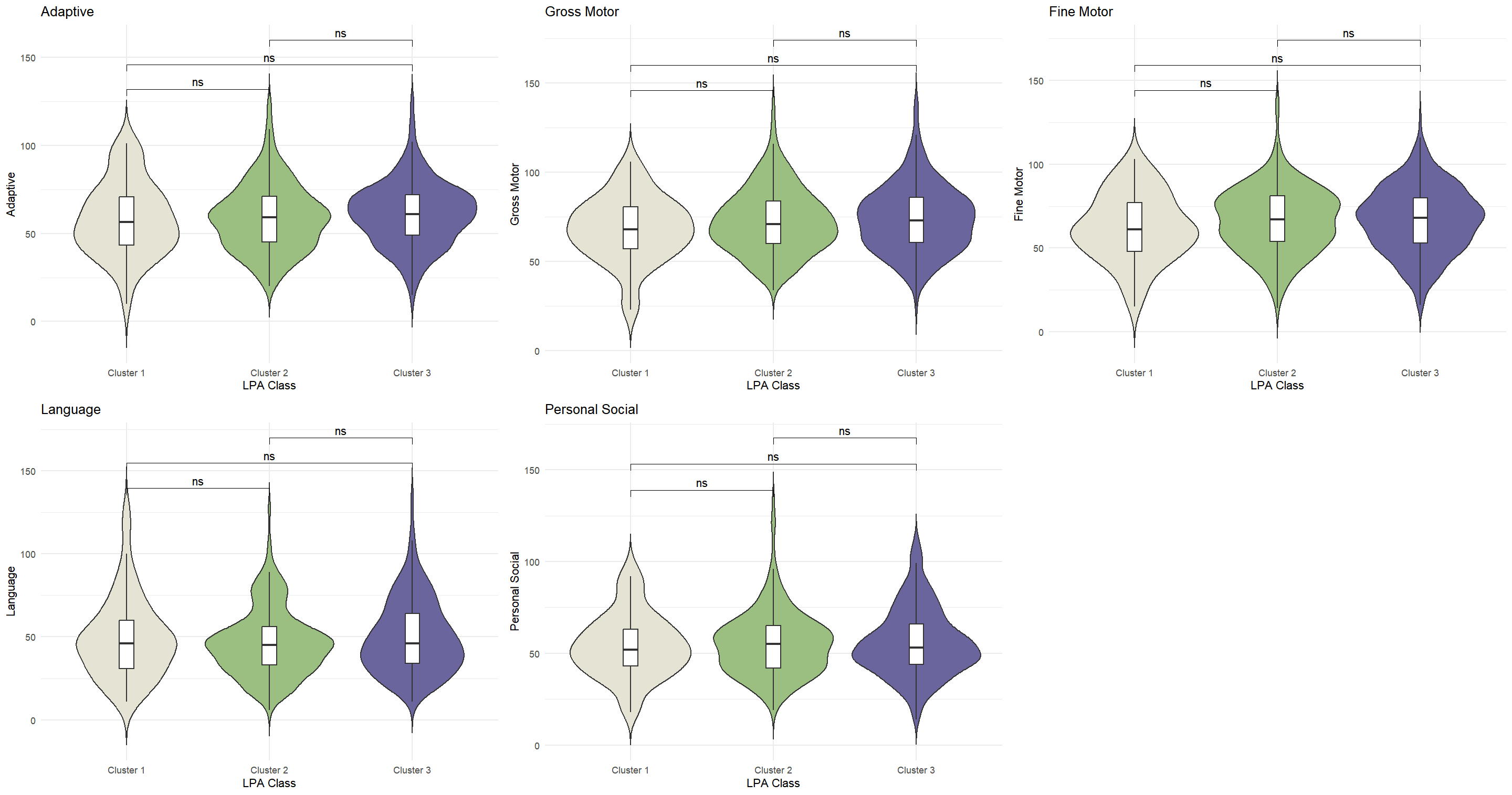


B
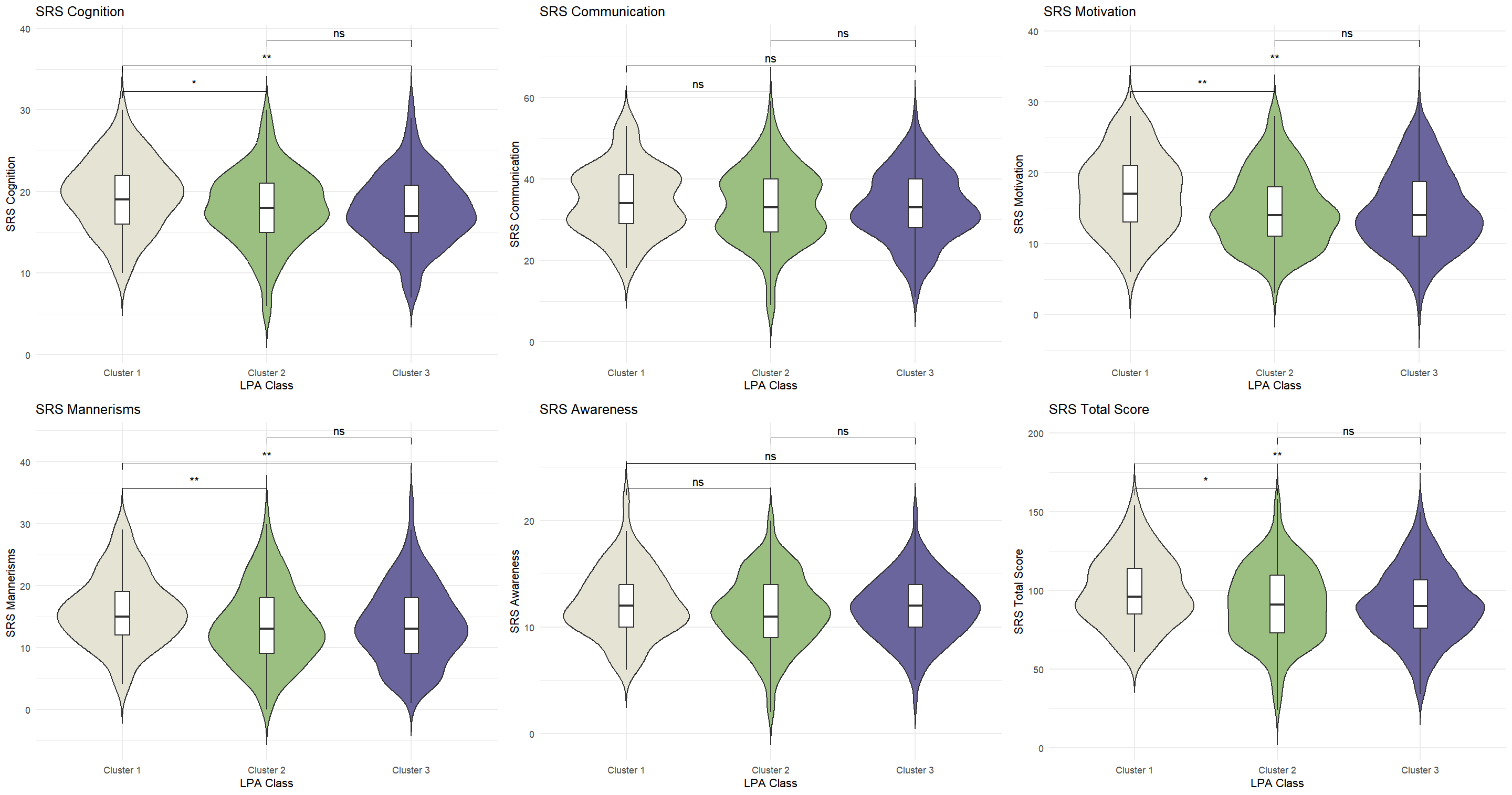


C
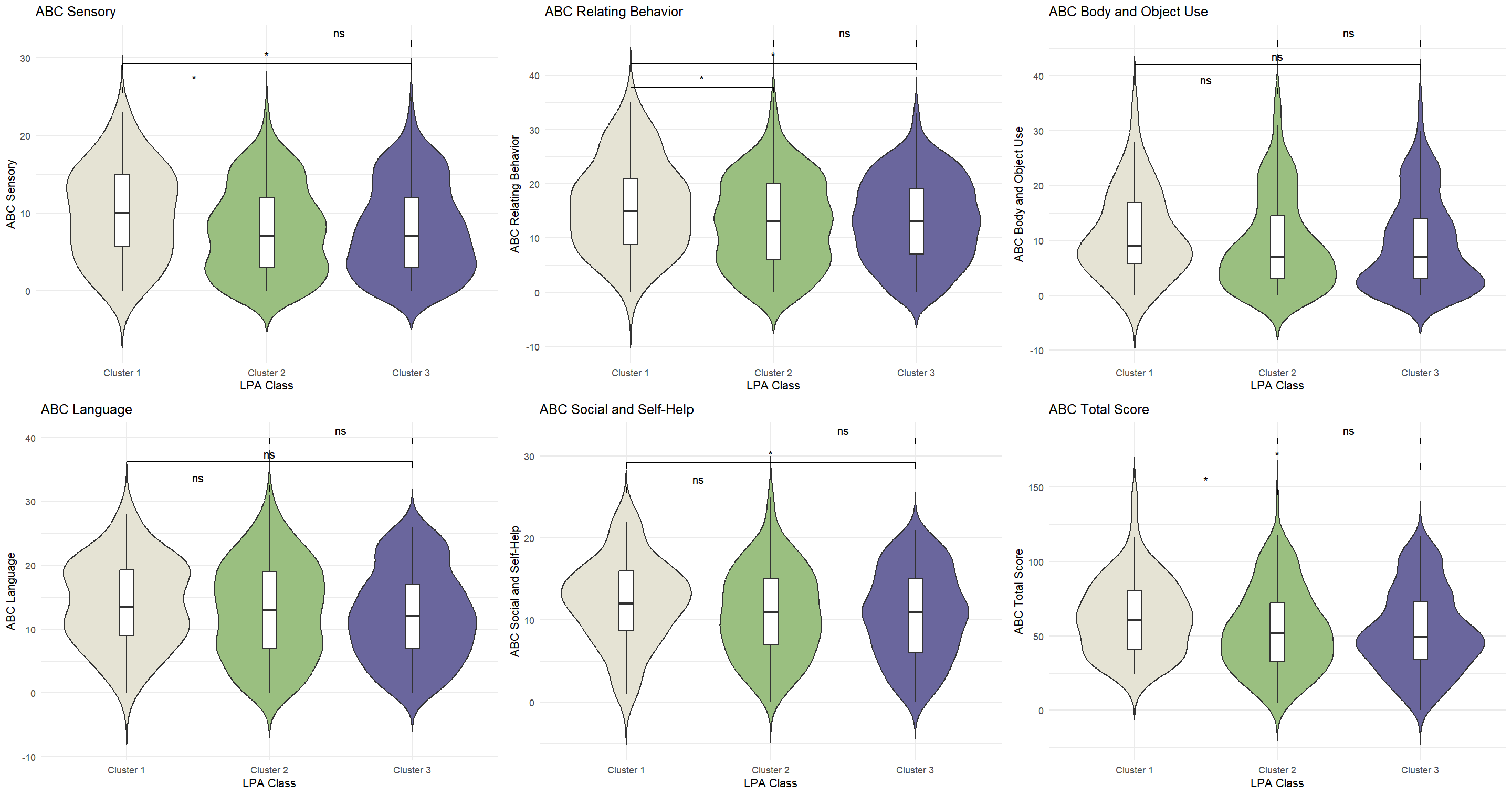


Supplementary Figure 3. Developmental and Behavioral Assessment across Different Sleep Subtypes in Children with Autism Spectrum Disorders: A. Gesell Developmental Scale ; B. Social Responsiveness Scale (SRS); C. Autism Behavior Checklist (ABC)

| **Supplementary Table 1.** Comparison of CSHQ Domain Scores Between Baseline and One-Year Follow-up Across Three ASD Sleep Subtypes | | | |
| --- | --- | --- | --- |
| Characteristics | LPA Group | *P*-value | Adjusted *P*-value |
| Bedtime Resistance | Cluster 1 | 0.233 | 0.466 |
| Sleep Onset Delay | Cluster 1 | 0.129 | 0.388 |
| Sleep Duration | Cluster 1 | 1 | 1 |
| Sleep Anxiety | Cluster 1 | 0.073 | 0.348 |
| Night Wakings | Cluster 1 | 0.072 | 0.348 |
| Parasomnias | Cluster 1 | 0.773 | 0.912 |
| Sleep Disordered Breathing | Cluster 1 | 0.182 | 0.407 |
| Daytime Sleepiness | Cluster 1 | 0.484 | 0.867 |
| Bedtime Resistance | Cluster 2 | 0.187 | 0.407 |
| Sleep Onset Delay | Cluster 2 | 0.506 | 0.867 |
| Sleep Duration | Cluster 2 | 0.836 | 0.912 |
| Sleep Anxiety | Cluster 2 | 0.829 | 0.912 |
| Night Wakings | Cluster 2 | 1 | 1 |
| Parasomnias | Cluster 2 | 0.129 | 0.388 |
| Sleep Disordered Breathing | Cluster 2 | 0.777 | 0.912 |
| Daytime Sleepiness | Cluster 2 | 0.091 | 0.366 |
| Bedtime Resistance | Cluster 3 | 0.035 | 0.284 |
| Sleep Onset Delay | Cluster 3 | 0.802 | 0.912 |
| Sleep Duration | Cluster 3 | 0.829 | 0.912 |
| Sleep Anxiety | Cluster 3 | 0.035 | 0.284 |
| Night Wakings | Cluster 3 | 0.766 | 0.912 |
| Parasomnias | Cluster 3 | 0.003 | 0.08 |
| Sleep Disordered Breathing | Cluster 3 | 0.149 | 0.397 |
| Daytime Sleepiness | Cluster 3 | 0.79 | 0.912 |
